# Extended-Spectrum β-Lactamase–Producing *Enterobacteriaceae* in a Malian Orphanage

**DOI:** 10.3201/eid1503.071637

**Published:** 2009-03

**Authors:** Didier Tandé, Nelle Jallot, Flabou Bougoudogo, Tracey Montagnon, Stéphanie Gouriou, Jacques Sizun

**Affiliations:** Brest University Hospital, Brest, France (D. Tandé, N. Jallot, T. Montagnon, S. Gouriou, J. Sizun); Bretagne Occidentale University, Brest (D. Tandé, S. Gouriou); National Institute of Research in Public Health, Bamako, Mali (F. Bougoudogo)

**Keywords:** Enterobacteria, extended-spectrum β-lactamases, orphanage, Mali, Africa, dispatch

## Abstract

We show high rates of extended-spectrum β-lactamase–producing *Enterobacteriaceae* carriage among the staff and children at an orphanage in Bamako, Mali. *Enterobacteriaceae* colonized in 100% and 63%, respectively, of the 38 children and 30 adults studied. Use of antimicrobial drugs appeared excessive and inappropriate; decontamination and hygiene protocols were also questioned.

Extended-spectrum β-lactamase (ESBL)–producing *Enterobacteriaceae* pose a major health problem because the incidence rate of infection is particularly high (*1*–*3*), and delays in the prescription of appropriate antimicrobial drug therapy for these infections are a risk factor for poor prognosis and death (*4*). In the 36 months preceding our study, children adopted by French families, and who were followed up at the Brest University Hospital, were shown to be carriers of ESBL-producing *Enterobacteriaceae*. All of these children had resided at the state orphanage in Bamako, Mali.

The dominant, and consequently widespread, manifestation of ESBL-producing *Enterobacteriaceae* in these children was the catalyst for our prospective investigation. We studied the carriage rate of these bacteria for the children and personnel of the orphanage and also the living conditions within the orphanage.

## The Study

This prospective cross-sectional study was conducted during September 10–19, 2003. The study population included 44 staff members and 39 children who were residing in the orphanage on the day the bacteriologic samples were taken. Adults were included on a voluntary basis and gave informed consent. Informed consent for the children was obtained from their legal representative, the director of the orphanage. Anonymity of participants was guaranteed.

We examined clinical data from charts as well as lifestyle habits, including personal and environmental hygiene. Environmental samples and children’s stool samples were obtained. Adults obtained their own stool samples. Environmental samples comprised potable tap water (1 sample) and surface samples (2 silicone bottle nipples, 1 child toilet, 2 faucets, and 1 sink that served as both a dishwashing and child-bathing station).

Samples were stored at 4°C and were sent by air within 18 hours to the microbiology laboratory of Brest University Hospital for analysis. Immediately upon arrival, the samples were injected into a Drigalski medium (bioMérieux, Marcy l’Etoile, France) supplemented with ceftazidime at 2 mg/L. This medium allows for selective isolation of resistant gram-negative bacteria. Colonies that developed on this medium showed different shapes and were systematically identified by using the API20E system (bioMérieux). Antimicrobial-drug susceptibility patterns were determined by the disk-diffusion method, as defined by the Committee for Antimicrobial Susceptibility Testing of the French Society for Microbiology (*5*)*.* *Enterobacteriaceae* were considered ESBL producers if synergy between third-generation cephalosporins and amoxicillin associated with clavulanic acid was detected (*5**,**6*)*.*

Of the 30 adults sampled, 19 (63%) were found to be colonized with 1–3 ESBL-producing *Enterobacteriaceae* of the same or different species (9 samples showed 1 strain; 7 showed 2 strains, and 3 showed 3 strains). More carriers were found in the caregiver adult group, who had direct contact with the children, than in those with less interactive responsibilities (e.g., administration and housekeeping staff), 90% (16/18) versus 25% (3/12), respectively (p<0.0016).

We included 38 of the 39 children in the analysis; no stool sample was available for the remaining child. All children sampled carried 1–3 ESBL-producing *Enterobacteriaceae* (21 with 2 strains and 9 with 3 strains). Bacteria samples from children were colonized more often than that of adults with >1 ESBL-producing *Enterobacteriaceae*, 78% (30/38) and 52% (10/19), respectively (p = 0.04). Water samples collected contained no ESBL-producing *Enterobacteriaceae*. The other 6 environmental samples testing positive showed 1 (4 samples), 2 (1 sample), or 3 (1 sample) strains of ESBL-producing *Enterobacteriaceae*.

We isolated 118 strains of ESBL-producing *Enterobacteriaceae*: *Escherichia coli* (56%), *Klebsiella pneumoniae* (36%), *K. oxytoca* (4%), and *Citrobacter freundii* (4%). Depending on the species, 36%–79% of strains were resistant to aminoglycosides, 20%–50% were resistant to fluoroquinolones, and >94% were resistant to cotrimoxazole.

Clonal relatedness of the 52 *E. coli* ESBL-producing strains was assessed by pulsed-field gel electrophoresis (Figure). Among these, 12 unique subtypes and 15 clusters of 2–7 clonal strains were identified. Dissemination might follow an allodemic pattern, corresponding to the spread of multiple specific clones or genetic elements.

**Figure:**
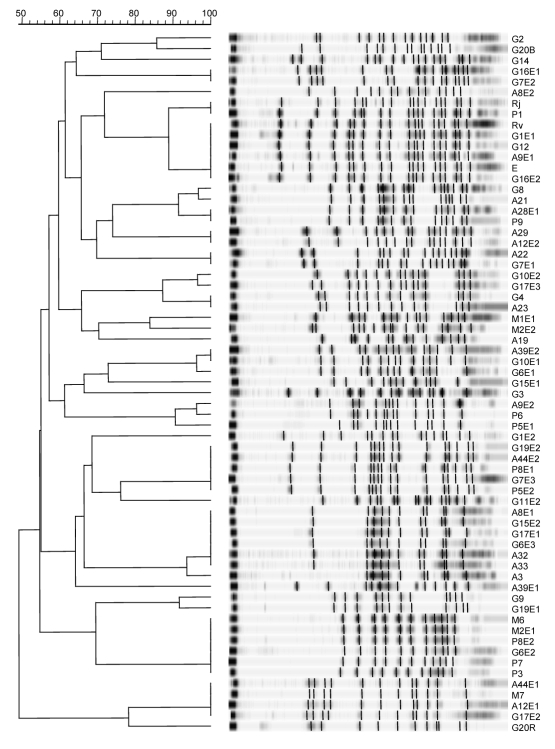
Representative *Xba*I pulsed-field gel electrophoresis profiles of extended-spectrum β-lactamase–producing *Escherichia coli*. Isolates denoted A originate from adults, P from children <4 months of age, M from children 4–12 months of age, G from children >12 months of age, and E and R from environmental samples.

The high colonization rate for caregiver adults within our study (90%) could logically and predominantly be attributed to direct contamination from the children. Furthermore, the excessive use of antimicrobial drugs also contributes to the emergence and spread of multidrug-resistant bacteria, as previously stated by Harbarth and van de Sande-Bruinsma (*7**,**8*)*.* The health records of the 39 children showed that 88 courses of antimicrobial drugs had been prescribed to 27 children during their stay at the orphanage during the 1,921 weeks that the children lived in the orphanage. Many of the antimicrobial drugs prescribed at the orphanage were used for infections of unlikely bacterial etiology. Many authors have emphasized that the inappropriate use of antimicrobial drugs, particularly among the children of various nonindustrialized nations, leads to antimicrobial resistance (*9**,**10*)*.* However, this phenomenon also exists in developing nations (*11*)*.* The antimicrobial drugs most frequently prescribed were third-generation cephalosporins and penicillins, which according to Colodner, favor the carriage of ESBL-producing *Enterobacteriaceae* (*12*)*.* The dosages prescribed were not always adapted to the precise weight of the children, thereby favoring the development of resistance. In addition, the duration of antimicrobial drug therapy, which also favors the development of resistance, was often limited for economic reasons, (*13**,**14*)*.*

We also suggest that dissemination of ESBL-producing *Enterobacteriaceae* within the orphanage might be explained by close contact between persons and massive contamination of the environment. With the exception of the water supply, all environmental samples tested positive despite compliance with posted hygiene rules and the best efforts and intentions of personnel. Feeding bottles were prepared on a bench top next to the sink, which was also used for bathing infants, and this sink was not regularly decontaminated during the day. Water was served in 1 shared drinking cup. Moreover, traditions such as eating from a shared plate and washing only 1 hand, along with a lack of material and financial means, appeared to clearly favor ESBL-producing *Enterobacteriaceae* colonization in the children. Childcare personnel washed their hands with soap and a diluted bleach solution before and after each caregiving interaction. Taps were operated manually without any particular contamination precautions. These environmental hygiene conditions contributed to the dissemination of the multidrug-resistant strains of *Enterobacteriaceae*.

Although this study gives a precise idea of the situation at the orphanage, our study has limitations. 1) The kinetics of colonization within the orphanage remain unknown; 2) the lack of a control group without ESBLs does not allow a comparison of the antimicrobial drug use; and 3) the rate of ESBL-producing *Enterobacteriaceae* carriage overall in Malian children of similar age and sex has not been scientifically determined.

## Conclusions

This study provides evidence that the carriage of ESBL-producing *Enterobacteriaceae* is prevalent at the orphanage in Bamako; dissemination is widespread among children and staff and within the environment. We identified 2 key factors responsible for this type of epidemic: 1) inadequate hygiene conditions favoring the spread of ESBL-producing *Enterobacteriaceae* in the environment and 2) the qualitative and quantitative inappropriate use of antimicrobial drugs. We therefore strongly recommend implementation of appropriate hygiene practices to limit colonization. Of equal importance is the coupling of these measures with a targeted and reasonable approach to the use of antimicrobial drugs.

Our results raise questions about the importance of quickly and accurately identifying persons who carry ESBL-producing *Enterobacteriaceae* because initiation of appropriate antimicrobial drug therapy may be delayed for infected patients and infection could spread rapidly. Testing for ESBL-producing *Enterobacteriaceae* in stools appears to be justified as part of the initial examination of all adopted children who have lived in an institution where these microorganisms are endemic.
